# [Corrigendum] Ethyl gallate suppresses proliferation and invasion in human breast cancer cells via Akt‑NF‑κB signaling

**DOI:** 10.3892/or.2024.8787

**Published:** 2024-07-31

**Authors:** Hongxia Cui, Jiaxin Yuan, Xiaohui Du, Ming Wang, Liling Yue, Jicheng Liu

Oncol Rep 33: 1284–1290, 2015; DOI: 10.3892/or.2014.3682

Following the publication of this article, an interested reader drew to the authors' attention that, for the cell migration assay data shown in Fig. 3C on p. 1287, the ‘2.5 μg/ml’ and ‘5.0 μg/ml’ panels appeared to be overlapping, such that these data were apparently derived from the same original source where they were intended to show the results from differently performed experiments. Upon asking the authors to provide an explanation, after having referred back to their original data, the authors realized that they had made an inadvertent error in assembling this figure.

The revised version of Fig. 3, now showing the correct data for the ‘5.0 μg/ml’ experiment, is shown on the next page. Note that the error made in assembling the data in Fig. 3 did not greatly affect either the results or the conclusions reported in this paper, and all the authors agree to the publication of this corrigendum. The authors regret that this error went unnoticed prior to the publication of their article, and are grateful to the Editor of *Oncology Reports* for granting them this opportunity to publish a corrigendum. They also apologize to the readership for any inconvenience caused.

## Figures and Tables

**Figure 3.**
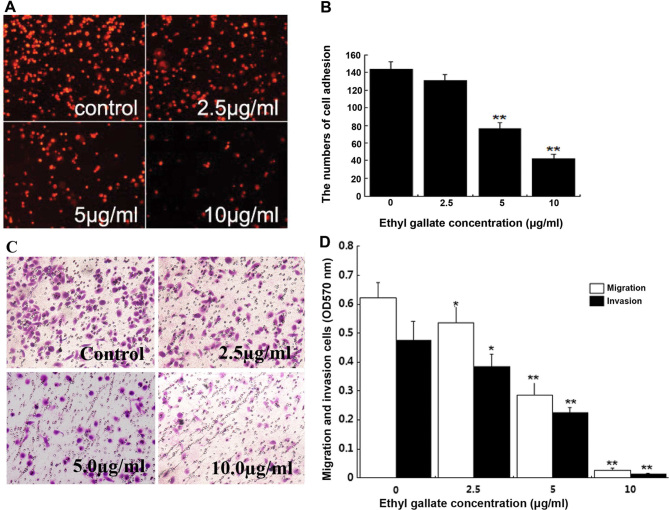
Ethyl gallate reduced cell adhesion, migration and invasion. (A) Images indicate the Dil-stained MDA-MB-231 cells that adhered to Matrigel were treated with Ethyl gallate for 2 h at indicated concentration. (B) Number of adhering cells were counted by fluorescence microscope and were shown as means ± SD. (C) Images indicate the crystal violet-stained MDA-MB-231 cells migrating through the Transwell chamber. The cells were treated for 2 h with 0 (as control) and 10 μg/ml. (D) Optical densities of migration and invasion were read at 570 nm and were shown as means ± SD. Statistical analysis was carried out using ANOVA. *P<0.05, **P<0.01 compared to the control.

